# Microglial activation protects against accumulation of tau aggregates in nondemented individuals with underlying Alzheimer’s disease pathology

**DOI:** 10.1038/s43587-022-00310-z

**Published:** 2022-11-28

**Authors:** Joana B. Pereira, Shorena Janelidze, Olof Strandberg, Christopher D. Whelan, Henrik Zetterberg, Kaj Blennow, Sebastian Palmqvist, Erik Stomrud, Niklas Mattsson-Carlgren, Oskar Hansson

**Affiliations:** 1 Department of Neurobiology, Care Sciences and Society, Division of Clinical Geriatrics, Karolinska Institute, Huddinge, Sweden; 2 Department of Clinical Sciences, Clinical Memory Research Unit, Lund University, Malmö, Sweden; 3 Biogen Research and Development, Cambridge, MA USA; 4 Institute of Neuroscience and Physiology, Department of Psychiatry and Neurochemistry, The Sahlgrenska Academy at the University of Gothenburg, Mölndal, Sweden; 5 Department of Neurodegenerative Disease, UCL Institute of Neurology, London, UK; 6 UK Dementia Research Institute at UCL, London, UK; 7 Clinical Neurochemistry Laboratory, Sahlgrenska University Hospital, Mölndal, Sweden; 8 Hong Kong Center for Neurodegenerative Diseases, Hong Kong, China; 9 Memory Clinic, Skåne University Hospital, Malmö, Sweden; 10 Department of Neurology, Skåne University Hospital, Lund University, Lund, Sweden; 11 Wallenberg Center for Molecular Medicine, Lund University, Lund, Sweden

**Keywords:** Predictive markers, Alzheimer's disease, Ageing

## Abstract

The role of microglia in tau accumulation is currently unclear but could provide an important insight into the mechanisms underlying Alzheimer’s disease (AD)^1^. Here, we measured the microglial marker soluble TREM2 and the disease-associated microglial activation stage 2 markers AXL, MERTK, GAS6, LPL, CST7, SPP1 and CSF1 in nondemented individuals from the Swedish BioFINDER-2 cohort who underwent longitudinal tau-positron emission tomography (PET), amyloid-PET and global cognitive assessment. To assess whether baseline microglial markers had an effect on AD-related changes, we studied three sub-groups of individuals: 121 with evidence of amyloid-PET pathology (A^+^), 64 with additional evidence of tau-PET pathology (A^+^T^+^) and 159 without amyloid- or tau-PET pathology (A^−^T^−^). Our results showed that increased levels of TREM2 were associated with slower amyloid accumulation in A^+^ individuals in addition to slower tau deposition and cognitive decline in A^+^T^+^ subjects. Similarly, higher levels of AXL, MERTK, GAS6, LPL, CST7 and CSF1 predicted slower tau accumulation and/or cognitive decline in the A^+^T^+^ group. These findings have important implications for future therapeutic strategies aiming to boost microglial protective functions in AD.

## Abstract (web summary)

The authors show that higher concentrations of disease-associated microglial activation stage 2 (DAM2) markers are associated with reduced tau accumulation and slower cognitive decline in nondemented individuals at risk for developing Alzheimer’s disease, suggesting that microglial activation delays disease progression.

## Main

It is now well recognized that microglia play a role in the development of AD^1^, mediating a wide range of mechanisms, including the phagocytosis of amyloid-β fibrils^2^. Genetic studies have also provided support of this view because loss-of-function mutations in the *TREM2* gene (encoding the microglia protein ‘triggering receptor expressed on myeloid cell 2’) increase the risk of developing AD dementia^3^. One possible reason is that microglia lacking functional TREM2 may be unable to transit to a disease-associated microglia stage 2 (DAM2), which allows the cells to sense tissue damage and restrict its spread^4,5^. In fact, TREM2 signaling is essential for the unique transcriptional signature that characterizes DAM2, including increases in the concentrations of, for example, TAM receptor tyrosine kinase (AXL) and MER proto-oncogene tyrosine kinase (MERTK), its ligand growth arrest specific 6 (GAS6), lipoprotein lipase (LPL), cystafin F (CST7), secreted phosphoprotein 1 (SPP1; also known as osteopontin) and the colony-stimulating factor 1 (CSF1)^6^, which could also contribute to the clearance of pathological protein aggregates^7^. Thus, DAM2 biomarkers might be an important therapeutic target for AD and their modulation may slow disease progression.

To our knowledge, no studies have yet assessed whether AXL, MERTK, GAS6, LPL, CST7, SPP1 or CSF1 is associated with reduced tau deposition in human individuals. The only studies that have been performed so far showed that higher levels of soluble TREM2 (sTREM2) in the cerebrospinal fluid (CSF) are associated with reduced amyloid-β accumulation^8,9^. However, the effects of microglial activation on future tau deposition are currently much less clear, with only one recent human study showing surprisingly harmful effects in a small sample over a short period of time^10^. These inconsistent results have led to some confusion in the field: is it possible that microglial activation ameliorates amyloid pathology and at the same time induces tau-dependent toxicity in AD? It is important to resolve this discrepancy considering that insoluble tau aggregates are more closely associated with neurodegeneration and cognitive impairment in AD^11^ and efficient immunomodulatory therapies will probably need to affect the accumulation of tau aggregates to provide a robust clinical benefit.

Thus, to address this important issue, in the present study, we assessed whether baseline sTREM2 as well as AXL, MERTK, GAS6, LPL, CST7, SPP1 and CSF1 concentrations are associated with future amyloid and tau accumulation and cognitive decline over several years in nondemented individuals at risk for AD due to the presence of amyloid- or tau-PET pathologies. Our underlying hypothesis was that higher DAM2 markers would correlate with reduced longitudinal amyloid and tau accumulation as well as better cognition in individuals at risk for AD, providing support for performing clinical trials with drugs that facilitate DAM2 activation.

To investigate this hypothesis, we measured the concentrations of sTREM2 in the CSF of 387 nondemented individuals from the Swedish BioFINDER-2 cohort who underwent longitudinal amyloid-PET (*n* = 259), tau-PET (*n* = 274) and cognitive assessment (*n* = 374). DAM2 markers were also measured in a subsample of this cohort (*n* = 344) (Table 1). To test our hypothesis that microglial markers protect against future AD-related changes in the presence of amyloid or tau, we studied two sub-groups based on previously established cut-offs for amyloid- and tau-PET positivity^12,13^: specifically 121 subjects with evidence of amyloid pathology (A^+^) and 64 with additional evidence of tau pathological changes (T^+^). The A^+^ group included both A^+^T^−^ and A^+^T^+^ individuals, whereas the T^+^ group included only A^+^T^+^ individuals because there were no subjects who were A^−^T^+^ in our study. The analyses with longitudinal amyloid-PET were conducted in subjects with amyloid pathology at baseline (A^+^), whereas those with tau-PET or cognition were performed in individuals with evidence of both amyloid and tau pathology (A^+^T^+^). To assess the specificity of our findings, we also conducted the analyses in 159 individuals without amyloid (A^−^) and tau (T^−^) burden.

**Table 1:** Characteristics of the sample

	A^−^T^−^ (*n* = 159)	A^+^ (*n* = 121)	A^+^T^+^ (*n* = 64)	A^−^T^−^ versus A^+^ (*P* value)	A^−^T^−^ versus A^+^T^+^ (*P* value)
Age (years)	74.0 (51.0–93.0)	73.0 (59.0–88.0)	74.0 (56.0–86.0)	0.006	<0.001
Sex (male:female)	79:80	60:61	2935	0.77	0.838
Education (years)	12.0 (7.0–26.0)	11.0 (7.0–22.0)	12.0 (6.0–33.0)	0.835	0.456
Cognitively impaired (%)	5	43	68.80	<0.001	<0.001
CSF sTREM2 (pg ml^−1^)	11.0 (4.6–23.3)	11.6 (4.7–29.6)	11.4 (5.53–21.94)	0.195	0.223
CSF AXL (pg ml^−1^)	2.0 (0.66–4.12)	2.14 (0.50–3.79)	1.96 (0.61–3.69)	0.236	0.144
CSF MERKT (pg ml^−1^)	−4.7 (−6.50–−3.01)	−4.6 (−7.14–−2.66)	−4.76 (−7.83–−2.80)	0.521	0.267
CSF GAS6 (pg ml^−1^)	2.5 (1.16–4.27)	2.8 (0.34–4.62)	2.6 (1.04–4.05)	0.189	0.096
CSF LPL (pg ml^−1^)	0.9 (−1.43–2.26)	1.01 (−1.68–2.50)	0.8 (−0.63 to 2.05)	0.037	0.068
CSF CST7 (pg ml^−1^)	−1.5 (−3.80–0.32)	1.4 (−3.75–1.36)	−1.4 (−2.96 to 0.09)	0.169	0.566
CSF SPP1 (pg ml^−1^)	3.3 (2.87 –3.80)	3.4 (2.83–3.80)	3.3 (2.91–3.65)	0.88	0.716
CSF CSF1 (pg ml^−1^)	1.9 (0.77–3.03)	2.0 (0.86–3.20)	1.88 (0.93–3.07)	0.228	0.148
Amyloid-PET global composite SUVR	0.9 (0.80–1.0)	1.4 (1.0–1.9)	1.63 (1.06–2.06)	<0.001	<0.001
Tau-PET I–II composite SUVR	(0.80–2.7)	1.3 (0.90–1.96)	1.83 (1.26–2.88)	<0.001	0.015
Tau-PET III–IV composite SUVR	1.2 (0.72–1.4)	1.2 (0.92–1.36)	1.79 (0.35–3.07)	<0.001	<0.001
Tau-PET V–VI composite SUVR	1.0 (0.63–1.23)	1.0 (0.78–1.17)	1.26 (1.01–1.85)	<0.001	<0.001
MMSE scores	29.0 (24.0–30.0)	28.0 (23.0–30.0)	27.0 (24.0–30.0)	0.011	0.187
Time to longitudinal amyloid-PET (years)					
Second scan	1.8 (1.17–3.98)	1.8 (0.15–2.56)	1.7 (1.04–1.98)	0.017	0.391
Third scan	3.86 (3.66–4.12)	3.73 (1.34–4.0)	3.6 (3.62–3.64)	0.023	0.075
Time to longitudinal tau-PET (years)					
Second scan	1.8 (0.74–3.90)	1.8 (0.64 − 2.46)	1.7 (0.78–1.96)	0.216	0.716
Third scan	1.8 (1.20–3.92)	1.8 (1.52–3.84)	1.7 (1.45–3.82)	0.549	0.942
Fourth scan	3.6 (1.52–3.72)	3.6 (3.50–3.65)	3.4 (3.44–3.46)	0.821	0.951
Time to longitudinal MMSE (years)					
Second evaluation	1.2 (0.94–2.11)	1.3 (0.40–1.99)	1.3 (0.40–1.75)	0.536	0.71
Third evaluation	2.0 (1.74–2.98)	2.2 (1.57–3.56)	2.2 (1.41–3.25)	0.03	0.165
Fourth evaluation	3.3 (2.84–3.55)	3.1 (2.76–3.56)	3.2 (2.70–4.20)	0.063	0.017
Fifth evaluation	4.0 (3.77–4.52)	4.0 (3.68–4.50)	4.2 (3.35–4.48)	0.702	0.538

Data presented in the table are reported as median (range) unless otherwise specified. *P* values were derived from *χ*^2^ tests for categorical measures and Kruskal–Wallis tests for continuous non-normally distributed measures. A^−^T^−^, subjects without amyloid-PET and tau-PET pathology; A^+^, all subjects with amyloid-PET pathology (A^+^T^−^ and A^+^T^+^); A^+^T^+^, subjects with both amyloid-PET and tau-PET pathology.

At baseline, there were significant positive correlations between amyloid burden and sTREM2 (*r* = 0.189, *P* = 0.012), AXL (*r* = 0.197, *P* = 0.009), CST7 (*r* = 0.191, *P* = 0.011) and CSF1 (*r* = 0.186, *P* = 0.014) in A^+^ individuals, but no associations with tau aggregates or cognition. We also observed significant correlations with sTREM2 when using CSF Aβ_42/40_ instead of amyloid-PET (*r* = −0.227, *P* = 0.002). This result is in line with evidence showing that the transcriptional signature of microglia becomes altered in the presence of amyloid plaques as a means of eliminating them through phagocytosis^2^.

To determine whether microglial markers were associated with longitudinal accumulation of amyloid and/or tau as well as cognitive decline, we tested separate linear mixed effect models using global amyloid-PET, three composite tau-PET regions (that is, for Braak stages I–II, Braak stages III–IV and Braak stages V–VI) and Mini-Mental State Examination (MMSE) scores as the outcomes, and the interactions between the microglial markers and time as predictors, while adjusting for age, sex, presence of cognitive impairment and years of education (for models including cognition). These analyses were corrected for multiple comparisons using false discovery rate (FDR); however, we also report uncorrected results (*P* < 0.05) in the text.

**Fig. 1:**
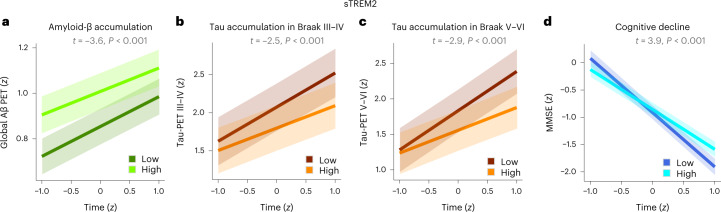
Higher sTREM2 levels are associated with lower amyloid and tau accumulation as well as cognitive decline in individuals with underlying AD pathology. **a**–**d**, Results of the linear mixed effect models showing that nondemented A^+^ individuals with higher baseline sTREM2 levels show less longitudinal accumulation of global amyloid (*n* = 115) (**a**), whereas A^+^T^+^ individuals show less tau aggregates in III–IV (**b**) and V–VI (**c**) Braak regions (*n* = 38) as well as a decline in the MMSE test scores (*n* = 53) (**d**). Amyloid (A^+^) and tau (T^+^) pathology were assessed using PET. All variables were *z*-transformed (*Z*), and the results were adjusted for multiple comparisons using FDR corrections (two tailed, *q* < 0.05). Data are presented as mean ± s.e.m.

With regard to sTREM2, our models showed that higher baseline levels predicted lower longitudinal amyloid accumulation in A^+^ individuals (Fig. 1a), in agreement with two recent in vivo studies showing the protective effects of this marker against amyloid deposition as measured with amyloid-PET^8,9^. Importantly, higher sTREM2 was also associated with lower accumulation of tau aggregates in Braak III–IV and V–VI tau-PET regions in A^+^T^+^ individuals (Fig. 1b,c). Finally, greater levels of sTREM2 predicted a less severe MMSE decline in A^+^T^+^ subjects (Fig. 1d).

**Fig. 2:**
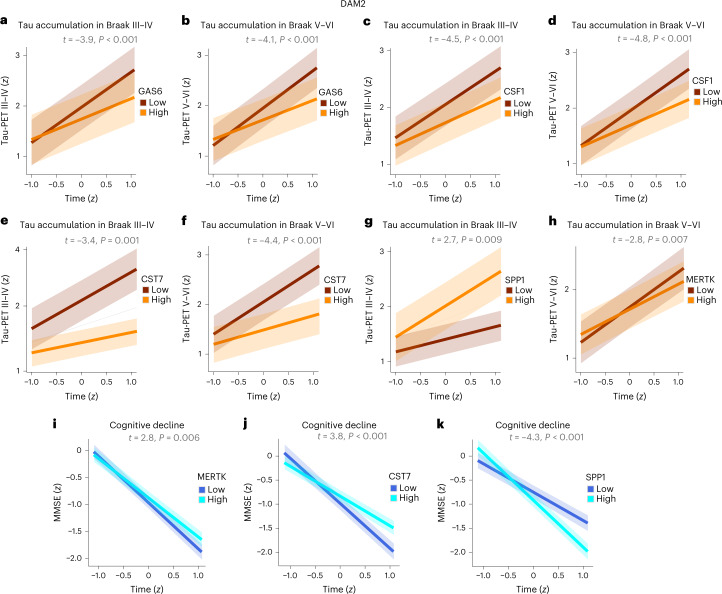
Higher DAM2 markers protect against future tau accumulation and cognitive decline in individuals with AD pathology. **a**–**k**, Results of the linear mixed effect models showing that nondemented A^+^T^+^ individuals with higher baseline DAM2 marker levels show lower longitudinal accumulation of tau aggregates in III–IV (**a**,**c**,**e**,**g**) and V–VI (**b**,**d**,**f**,**h**) Braak regions (GAS6: *n* = 38; CSF1: *n* = 37; CST7: *n* = 37; SPP1: *n* = 38; MERKT: *n* = 37) as well as a decline in the MMSE test scores (MERKT: *n* = 51; CST7: *n* = 51) (**i**,**j**), except for the SPP1 DAM2 marker, which showed the opposite results (*n* = 52) (**g**,**k**). Amyloid (A^+^) and tau (T^+^) pathology were assessed using PET. All variables were *z*-transformed (*Z*), and the results were adjusted for multiple comparisons using FDR corrections (two tailed, *q* < 0.05). Data are presented as the mean ± s.e.m.

As we obtained interesting results with sTREM2, which is associated with the transition of microglia to DAM2, we then proceeded to investigate the DAM2 markers in relation to the outcomes of interest. In contrast to sTREM2, none of the DAM2 biomarkers was associated with longitudinal amyloid-PET changes. Instead, we found that higher GAS6, CSF1 and CST7 were associated with slower deposition of insoluble tau aggregates in neocortical regions, that is, in Braak III–IV (Fig. 2a,c,e,g) as well as in Braak V–VI regions (Fig. 2b,d,f,h), after FDR corrections. At an uncorrected level, there were also significant associations between Braak III–IV regions and AXL (*t* = −2.6, *P* = 0.014), MERTK (*t* = −2.6, *P* = 0.014) and LPL (*t* = −2.2, *P* = 0.031), as well as between Braak V–VI regions and AXL (*t* = −2.4, *P* = 0.023) and LPL (*t* = −2.5, *P* = 0.018).

Next, we studied whether the associations between higher levels of DAM2 markers and slower accumulation of tau aggregates were independent of changes in amyloid accumulation, which would indicate amyloid-independent effects of microglia on tau aggregation. We found that all associations between TREM2 and the other DAM2 markers were still significantly associated with tau-PET signal changes in Braak III–IV and V–VI regions when longitudinal amyloid-PET changes were added as an additional covariate (all *P* < 0.05).

When using change in cognition as an outcome, we found that MERTK and CST7 predicted less pronounced cognitive decline over time in A^+^T^+^ individuals after FDR corrections (Fig. 2i,j). Moreover, at an uncorrected level, GAS6 (*t* = 2.2, *P* = 0.030) and CSF1 (*t* = 2.3, *P* = 0.021) also predicted lower cognitive decline. In additional analyses assessing whether age or sex interacted with microglial markers, we observed that sex showed significant interactions with CST7 (*t* = 3.4, *P* < 0.001) and MERTK (*t* = 4.0, *P* < 0.001) in A^+^ individuals in addition to interactions with TREM2 (*t* = 3.9, *P* < 0.001), CSF1 (*t* = 3.3, *P* = 0.001), CST7 (*t* = 3.6, *P* < 0.001) and MERTK (*t* = 3.9, *P* < 0.001) in A^+^T^+^ individuals, indicating that women with higher baseline microglial markers showed less steep cognitive decline compared with men.

It is interesting that one DAM2-associated marker, SPP1, showed the opposite results to all other DAM2 markers by predicting faster tau accumulation in Braak III–IV (Fig. 2g), as well as faster cognitive decline (Fig. 2k) in A^+^T^+^ individuals. Similar results were found at an uncorrected level between SPP1 and Braak V–VI regions (*t* = 2.671, *P* = 0.01). These results suggest that not all microglial activation is beneficial and that future studies should investigate different DAM2 markers and their protective as well as their detrimental effects.

No statistically significant relationships were found for any of the analyses in A^−^T^−^ subjects, confirming the specificity of our results.

**Extended Data Fig. 1:**
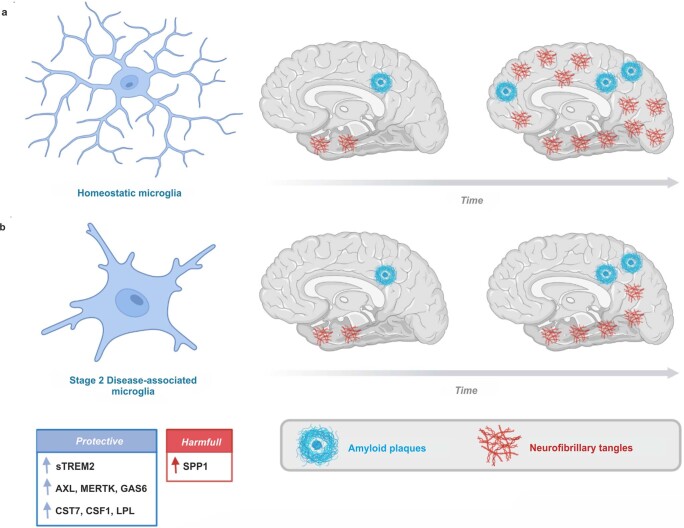
Visual representation of the role of microglia activation in longitudinal accumulation of amyloid and tau aggregates. When microglia are in a homeostatic state, they do not protect against the accumulation of amyloid and tau aggregates, which increase over time in individuals at risk of developing Alzheimer’s disease (AD) (**a**). In contrast, when sTREM2 signaling is present, this marker allows microglia to transition from an homeostatic state to a disease-associated microglia stage 2 (DAM2) state characterized by higher AXL, MERTK, GAS6, CST7, CSF1 and LPL levels. The increases in sTREM2 and DAM2 markers have protective effects by reducing the longitudinal deposition of amyloid plaques and tau aggregates (**b**). An important exception is the DAM2 marker SPP1, which seems to have a detrimental rather than a protective effect on future AD-related changes. This figure was created using the Biorender.com software.

There is an urgent need for new, more comprehensive approaches to treat AD because targeting any single process such as amyloid-β or tau accumulation, even if successful, may not be sufficient on its own to slow down the progression of the disease. Thus, boosting an intrinsic protective mechanism such as DAM2 activation might provide important benefits^14,15^. Specifically, we found that, in contrast to previous preliminary findings in a small longitudinal sample of individuals^10^, increases in TREM2, AXL, MERTK, GAS6, LPL, CST7 and CSF1 are associated with less severe AD-related changes, indicating that microglia may strengthen the brain’s resilience to pathological processes (Extended Data Fig. 1a,b).

It is interesting that the DAM2 markers were associated with slower tau accumulation only outside the medial temporal lobe. In other words, microglial activation slowed down the neocortical Braak III–VI stages of the disease, where tau accumulation is clearly dependent on cortical amyloid-β aggregates and is strongly associated with the development of dementia. These findings agree with recent animal models exhibiting amyloid-β pathology that showed a protective role of microglial activation in reducing the accumulation of insoluble tau aggregates^16–18^. Still, we found that the slowing of tau accumulation associated with DAM2 was independent of longitudinal changes in amyloid. Thus, DAM2 seems to have direct protective effects in AD by reducing tau accumulation in the neocortex. This agrees with recent findings in experimental models showing that human pathological tau might be degraded less efficiently in macrophages lacking TREM2, indicating that DAM2 might have an important role in removing tau seeds^17^.

Contrary to our findings, several previous animal studies have shown that microglial activation has detrimental effects in the brain by inducing toxic neuroinflamation^19^. This discrepancy between some of the previous results and ours is probably due to the complexity of microglial phenotypes, which can vary depending on the specific cell environment and disease stage^20–22^. For instance, animal studies have shown that TREM2-dependent microglial functions limit amyloid plaque growth during early but not late disease stages^23^. Moreover, the way that TREM2 signaling affects the formation of amyloid plaques may depend on animal model, sex, brain region and detection method^20,23–26^, even though there is accumulating evidence showing that reduced TREM2 signaling results in amyloid-β plaques with less compacted morphology and more damaged neurites surrounding the plaques^25,27,28^. In contrast to amyloid-β, fewer experimental studies have focused on the effects of microglia on the accumulation of tau pathology. For instance, two recent studies have shown that reduced TREM2 signaling facilitates the accumulation and spread of tau in mice models developing amyloid-β plaques, but not in tau models without amyloid-β pathology^16,18^. Thus, microglia could have different roles by exerting protective and detrimental effects on amyloid-β and tau pathology depending on, for example, animal model and disease stage. Future studies are needed to disentangle the dual role of microglia and establish the correspondence between microglial phenotype with disease stage and TREM2 deficiency.

Some limitations should be recognized in our work. Although the total sample size was large, with approximately 400 individuals with multiple biomarkers and measures, the number of cases in the A^+^T^+^ sub-group of 64 individuals was considerably smaller, indicating that our results should be replicated in larger sub-groups. Moreover, our longitudinal PET and cognitive measures were available for a period of only 4 years, but we are currently in the process of acquiring these measures over a larger time period for the same individuals included in the present study. Finally, it would have been interesting to include a microglial PET tracer in the present study to compare it with the CSF biomarkers, something that should be addressed in future studies.

Taken together, our findings in humans support a role of DAM2 in mitigating neocortical accumulation of insoluble tau aggregates, which place microglial activation in the center of the amyloid cascade hypothesis for AD^29,30^, suggesting that it should be taken into account in future AD therapies^14,15^.

## Methods

### Participants

The present study included 387 nondemented individuals from the prospective Swedish BioFINDER-2 cohort (NCT03174938), which has the aim of identifying new biomarkers for the diagnosis of AD and other neurodegenerative diseases at an early stage. All participants were recruited between 2017 and 2020 and included cognitively normal individuals, subjects with subjective cognitive decline (SCD) and patients with mild cognitive impairment (MCI). Cognitively normal individuals were recruited from two population-based studies in Malmö, Sweden, that is, the Malmö Diet and Cancer study and the Malmö Offspring study^31,32^. Cognitively normal subjects were required to: (1) be aged between 45 and 100 years; (2) not show cognitive symptoms evaluated by a physician with extensive experience in cognitive disorders; (3) present a score on the MMSE that is between 26 and 30 (older participants) or 27 and 30 (younger participants); (4) not fulfill mild or major neurocognitive disorder criteria following the *Diagnostic and Statistical Manual of Mental Disorders*, 5th edn (DSM-5)^33^ guidelines; and (5) be fluent in Swedish. Participants with SCD or MCI were recruited from the Skåne University Hospital and Hospital of Ängelholm in Sweden^34^, and they were were required to: (1) be aged between 40 and 100 years; (2) have been referred to the memory clinic due to the presence cognitive symptoms; (3) have a score on the MMSE that is between 24 and 30 points; (4) not fulfill any dementia criteria following DSM-5 guidelines; and (5) be fluent in Swedish. Participants with SCD were deemed cognitively normal in agreement with the National Institute on Aging–Alzheimer’s Association research framework^35^. Participants were considered to have MCI if their performance in any cognitive domain was below −1.5 s.d. based on age and education categories test norms.

The study was approved by the Radiation Safety Committee of Skåne University Hospital, the Swedish Medical and Products Agency and the Regional Ethical Review Board of Lund University in Sweden. All participants provided written, informed consent following the Declaration of Helsinki guidelines. No compensation was provided to any participant.

### CSF biomarkers

CSF samples were collected by lumbar puncture and stored at −80 °C in LoBind poly(propylene) tubes. An Elecsys assay using the NeuroToolKit robust prototype (Roche Diagnostics) was used to determine the concentrations of sTREM2 (ref. ^36^). Concentrations of AXL, MERTK, GAS6, LPL, CST7, SPP1 and CSF1 were quantified using the Olink Explore 3072 platform, developed by Olink Proteomics^37^. The measurements of the proteins were performed using technology based on proximity extension assay, in accordance with the protocol of the manufacturer^24^. First, the antigens were incubated with pairs of antibodies that included DNA oligonucleotides bound to each of the proteins that we wanted to measure. Oligonucleotides in close proximity were used to create a template for hybridization and extension, and PCR was used for preamplification. Specific primers were digested on a real-time quantitative PCR chip after the digestion of residual primers and using a Biomark HD Instrument. The proteins were quantified as normalized protein expression log_2_ scale. A few subjects were excluded from the statistical analyses of AXL (*n* = 3), MERTK (*n* = 4), GAS6 (*n* = 2), LPL (*n* = 4), CST7 (*n* = 11), SPP1 (*n* = 2) and CSF1 (*n* = 11) due to low assay quality.

### Imaging acquisition and preprocessing

Participants underwent [^18^F] RO948 PET and [^18^F]flutemetamol PET on General Electrics Discovery MI scanners as well as structural magnetic resonance imaging on a Siemens Prisma 3T scanner. The [^18^F] RO948 PET scans were acquired 70–90 min after an injection of 370 MBq of [^18^F] RO948. The [^18^F]flutemetamol PET images were acquired 90–110 min after an injection of 185 MBq of [^18^F]flutemetamol. Structural T1-weighted images were collected using a magnetization-prepared rapid gradient echo sequence with 178 slices, repetition and echo times of 1,950 ms and 3.4 ms, a flip angle of 9°, an inversion time of 900 ms and a spatial resolution of 1 mm^3^. All PET images were submitted to different preprocessing steps that included motion correction, time averaging and coregistration to their corresponding skull-stripped, longitudinally preprocessed, T1-weighted images with FreeSurfer (v.6.0; https://surfer.nmr.mgh.harvard.edu). The [^18^F] RO948 scans were normalized by the inferior cerebellar gray matter, and [^18^F]flutemetamol scans were normalized by the whole cerebellum.

### Longitudinal imaging analyses

To examine longitudinal changes in amyloid deposition on [^18^F]flutemetamol PET and tau accumulation on [^18^F] RO948 PET, we used regions of interest. For [^18^F]flutemetamol images, we calculated the standard uptake value ratio (SUVR) for a global composite region as defined in a previous study^12^. For [^18^F] RO948 PET images, we measured the SUVRs of three previously defined composite regions corresponding to Braak stages I–II, III-–IV and V–VI, as defined in another previous study^25^. Subjects were classified as having amyloid pathology (A^+^) if their global amyloid-PET SUVR exceeded 1.033 and with tau pathology (T^+^) if their I–IV tau-PET SUVR exceeded 1.34 based on previously established cut-offs^38,39^.

### Statistical analyses

To test whether baseline sTREM2, AXL, MERTK, GAS6, LPL, CST7, SPP1 or CSF1 levels were associated with longitudinal brain imaging and cognitive changes, we used linear mixed effect models in R (v.3.5.1). These models were conducted separately in A^−^T^−^, A^+^ and A^+^T^+^ groups and included global amyloid-PET SUVR, tau-PET SUVR (I–II, III–IV, V–VI) or global cognition (MMSE) as dependent variables and the CSF microglial markers, time, age, sex, presence of cognitive impairment and years of education (for cognition) as fixed effects. In these analyses the interaction between biomarker levels and time as well as the main effects and random effects for intercepts were also included. We ran separate models for each outcome and each microglial marker.

All the analyses were corrected for multiple comparisons using FDR (*q* < 0.05, two tailed); however, we also report uncorrected results that were significant at *P* < 0.05 in the text.

### Reporting summary

Further information on research design is available in the Nature Portfolio Reporting Summary linked to this article.

### Supplementary information

Reporting Summary

## Data Availability

Data in anonymized format will be shared upon request with the aim of replicating the results and procedures shown in the article while ensuring that the transfer of data agrees with EU legislation, Region Skåne and the Ethical Review Board of Sweden, and should be coordinated through a material transfer agreement.

## Code availability

The code used in the present study is simple and well documented in the literature, but will be shared on request from a qualified academic investigator.
