# Supplementary material for: Microglial activation protects against accumulation of tau aggregates in nondemented individuals with underlying Alzheimer’s disease pathology
Source: Nat Aging. 2022 Nov 28;2(12):1138–44. doi: 10.1038/s43587-022-00310-z (PMC10154192; doi:10.1038/s43587-022-00310-z)
Supplement: Supplementary file 1 — Reporting Summary [file 43587_2022_310_MOESM1_ESM.pdf]

nature portfolio

Corresponding author(s): Joana B. Pereira, Oskar Hansson

Last updated by author(s): Sep 28, 2022

Reporting Summary

Nature Portfolio wishes to improve the reproducibility of the work that we publish. This form provides structure for consistency and transparency in reporting. For further information on Nature Portfolio policies, see our Editorial Policies and the Editorial Policy Checklist.

Statistics

For all statistical analyses, confirm that the following items are present in the figure legend, table legend, main text, or Methods section.

n/a	Confirmed	
☐	☒	The exact sample size (*n*) for each experimental group/condition, given as a discrete number and unit of measurement
☐	☒	A statement on whether measurements were taken from distinct samples or whether the same sample was measured repeatedly
☐	☒	The statistical test(s) used AND whether they are one- or two-sided<br>*Only common tests should be described solely by name; describe more complex techniques in the Methods section.*
☐	☒	A description of all covariates tested
☐	☒	A description of any assumptions or corrections, such as tests of normality and adjustment for multiple comparisons
☐	☒	A full description of the statistical parameters including central tendency (e.g. means) or other basic estimates (e.g. regression coefficient) AND variation (e.g. standard deviation) or associated estimates of uncertainty (e.g. confidence intervals)
☐	☒	For null hypothesis testing, the test statistic (e.g. *F*, *t*, *r*) with confidence intervals, effect sizes, degrees of freedom and *P* value noted<br>*Give P values as exact values whenever suitable.*
☒	☐	For Bayesian analysis, information on the choice of priors and Markov chain Monte Carlo settings
☒	☐	For hierarchical and complex designs, identification of the appropriate level for tests and full reporting of outcomes
☒	☐	Estimates of effect sizes (e.g. Cohen's *d*, Pearson's *r*), indicating how they were calculated

Our web collection on statistics for biologists contains articles on many of the points above.

Software and code

Policy information about availability of computer code

Data collection	An Elecsys assay using the NeuroToolKit robust prototype (Roche Diagnostics) was used to determine the concentrations of sTREM235. Concentrations of AXL, MERTK, GAS6, LPL, CST7, SPP1 and CSF1 concentrations were quantified using the Olink® Explore 3072 platform, developed by Olink Proteomics (Uppsala, Sweden). Participants underwent 18F-RO948 PET and 18F-flutemetamol PET on General Electrics Discovery MI scanners as well as structural MRI on a Siemens Prisma 3T scanner.
Data analysis	We used the FreeSurfer software (version 6.0, https://surfer.nmr.mgh.harvard.edu/) for defining regions of interest to quantify amyloid and tau PET burden. The statistical analyses were performed using linear mixed effect models in R (version 3.5.1).

For manuscripts utilizing custom algorithms or software that are central to the research but not yet described in published literature, software must be made available to editors and reviewers. We strongly encourage code deposition in a community repository (e.g. GitHub). See the Nature Portfolio guidelines for submitting code & software for further information.

Data

Policy information about availability of data

All manuscripts must include a data availability statement. This statement should provide the following information, where applicable:

- Accession codes, unique identifiers, or web links for publicly available datasets
- A description of any restrictions on data availability
- For clinical datasets or third party data, please ensure that the statement adheres to our policy

Anonymized data will be shared by request from a qualified academic investigator (Prof. Oskar Hansson, Oskar.Hansson@med.lu.se) for the sole purpose of replicating procedures and results presented in the article and providing that the data transfer is in agreement with EU legislation on the general data protection regulation and decisions by the Ethical Review Board of Sweden and Region Skåne, which should be regulated in a material transfer agreement. The time frame for responses to requests is 2 months.

Human research participants

Policy information about studies involving human research participants and Sex and Gender in Research.

Reporting on sex and gender	Sex information is available on Table 1. In addition, some results stratified by sex are also reported: "In additional analyses assessing whether age or sex interacted with microglial markers, we observed that sex showed significant interactions with CST7 (t=3.4, P<0.001) and MERTK (t=4.0, P<0.001) in A+ individuals in addition to interactions with TREM2 (t=3.9, P<0.001), CSF1 (t=3.3, P=0.001), CST7 (t=3.6, P<0.001) and MERTK (t=3.9, P<0.001) in A+T+ individuals, indicating that women with higher baseline microglial markers showed less steep cognitive decline compared to men."
Population characteristics	We included age and sex in all statistical analyses examining the association between baseline microglial markers and longitudinal amyloid and tau PET pathology, whereas the analyses involving longitudinal cognition were adjusted by age, sex and education. The study included included cognitively normal individuals, subjects with subjective cognitive decline (SCD) and patients with mild cognitive impairment (MCI).
Recruitment	Cognitively normal persons were recruited from two population-based studies in Malmö, Sweden, i.e. the Malmö Diet and Cancer study and the Malmö Offspring study, whereas participants with SCD or MCI were recruited from the Skåne University Hospital and Hospital of Ängelholm in Sweden. As in most research studies of this kind, the individuals that volunteered to participate in the BioFINDER-2 protocols had higher education and lower number of commorbidities.
Ethics oversight	The Regional Ethical Review Board of Lund University, the Swedish Medical and Products Agency, and the Radiation Safety Committee of Skåne University Hospital in Sweden approved the study and written, informed consent was obtained from all participants according to the Declaration of Helsinki.

Note that full information on the approval of the study protocol must also be provided in the manuscript.

Field-specific reporting

Please select the one below that is the best fit for your research. If you are not sure, read the appropriate sections before making your selection.

☒ Life sciences ☐ Behavioural & social sciences ☐ Ecological, evolutionary & environmental sciences

For a reference copy of the document with all sections, see nature.com/documents/nr-reporting-summary-flat.pdf

Life sciences study design

All studies must disclose on these points even when the disclosure is negative.

Sample size	Participants from the BioFinder cohort were included in the study if they were non-demented, had undergone lumbar puncture to determine the concentrations of microglial proteins in addition to longitudinal PET imaging and cognitive assessments. No power calculations were performed beforehand. The final sample size of 387 individuals was relatively large compared to previous similar studies so we believed it was sufficient to test our hypotheses.
Data exclusions	A few subjects were excluded from the statistical analyses of AXL (n = 3), MERTK (n = 4), GAS6 (n = 2), LPL (n = 4), CST7 (n = 11), SPP1 (n = 2) and CSF1 (n = 11) due to low assay quality.
Replication	No replication was performed in this study due to lack of other cohorts with the DAM2 markers assessed in the current study in combination with longitudinal tau PET imaging.
Randomization	The statistical models were conducted separately in A-T-, A+ and A+T+ groups and included global amyloid-PET SUVR, tau-PET SUVR (I-II, III-IV, V-VI), or global cognition (MMSE) as dependent variables and the CSF microglial markers, time, age, sex, presence of cognitive impairment, years of education (for cognition) as fixed effects.

Blinding: The investigators were blinded to group allocation during data acquisition but not during statistical analyses because the definition of groups and the models that were ran in each of them were performed by the same investigator (Joana B. Pereira). However, an independent investigator blinded to the group allocation was requested to repeat the analyses to ensure all the reported results were correct (Niklas Mattsson-Calgren).

Reporting for specific materials, systems and methods

We require information from authors about some types of materials, experimental systems and methods used in many studies. Here, indicate whether each material, system or method listed is relevant to your study. If you are not sure if a list item applies to your research, read the appropriate section before selecting a response.

Materials & experimental systems

n/a	Involved in the study
☒	☐ Antibodies
☒	☐ Eukaryotic cell lines
☒	☐ Palaeontology and archaeology
☒	☐ Animals and other organisms
☐	☒ Clinical data
☒	☐ Dual use research of concern

Methods

n/a	Involved in the study
☒	☐ ChIP-seq
☒	☐ Flow cytometry
☐	☒ MRI-based neuroimaging

Clinical data

Policy information about clinical studies
All manuscripts should comply with the ICMJE guidelines for publication of clinical research and a completed CONSORT checklist must be included with all submissions.

Clinical trial registration: N/A

Study protocol: Protocol: NCT03174938

Data collection: All participants were recruited Malmö, Sweden or the Skåne University Hospital and Hospital of Ängelholm in Sweden between 2017 and 2020 and included cognitively unimpaired individuals, subjects with subjective cognitive decline (SCD) and patients with mild cognitive impairment (MCI).

Outcomes: To test whether baseline sTREM2, AXL, MERTK, GAS6, LPL, CST7, SPP1 or CSF1 levels were associated with longitudinal changes in brain imaging and cognition we used linear mixed effect models in R (version 3.5.1). These models were conducted separately in A-T-, A+ and A+T+ groups and included global amyloid-PET SUVR, tau-PET SUVR (I-II, III-IV, V-VI), or global cognition (MMSE) as dependent variables and the CSF microglial markers, time, age, sex, presence of cognitive impairment, years of education (for cognition) as fixed effects. We also included the interaction between biomarker levels and time (together with the main effects), and random effects for intercepts. Separate models were tested for each outcome and each microglial marker.

Magnetic resonance imaging

Experimental design

Design type: Structural T1-weighted MRI.

Design specifications: No blocks or trials are applied during T1-weighted MRI imaging. This type of design details are usually done only for task-based functional MRI.

Behavioral performance measures: No behavioral measures are acquired during T1-weighted MRI imaging. These measures are usually acquired during task-based functional MRI.

Acquisition

Imaging type(s): Structural.

Field strength: 3 Tesla.

Sequence & imaging parameters: Structural T1-weighted images were acquired using a magnetization-prepared rapid gradient echo (MPRAGE) sequence using the following parameters: 178 slices, repetition time: 1950 ms, echo time: 3.4 ms, inversion time: 900 ms, flip angle: 9, 1 mm isotropic voxels.

Area of acquisition: Whole brain.

Diffusion MRI: ☐ Used ☒ Not used

Preprocessing

Preprocessing software	FreeSurfer (version 6.0, https://surfer.nmr.mgh.harvard.edu/).
Normalization	Linear and nonlinear transformations were applied.
Normalization template	Normalized using individual subject average across timepoints and then registered to Talairach space.
Noise and artifact removal	Images with motion artifacts were excluded from the BioFinder database before imaging preprocessing.
Volume censoring	Criteria for motion was > 1 mm3.

Statistical modeling & inference

Model type and settings	Linear mixed effects models.
Effect(s) tested	Interaction between time and biomarker levels in the prediction of imaging and cognitive outcomes.

Specify type of analysis: ☐ Whole brain ☒ ROI-based ☐ Both

Anatomical location(s)	Global composite region for amyloid PET uptake and Braak stage regions I-II, III-IV and V-VI for tau PET uptake.
Statistic type for inference (See Eklund et al. 2016)	$p < 0.05$
Correction	FDR corrections

Models & analysis

n/a	Involved in the study
☒	☐ Functional and/or effective connectivity
☒	☐ Graph analysis
☒	☐ Multivariate modeling or predictive analysis
